# Safety and effcacy of remimazolam tosilate for sedation during combined spinal-epidural anesthesia for orthopedic procedures: a randomized controlled trial

**DOI:** 10.1186/s12871-024-02451-7

**Published:** 2024-02-26

**Authors:** Yufei Chen, Yimeng Cai, Guoqing Yu, Xi Zhang, Tian Hu, Rui Xue

**Affiliations:** Department of Anesthesiology, Renmin Hospital,Hubei University of Medicine, Shiyan City, Hubei Province China

**Keywords:** Remimazolam, Sedation, Dexmedetomidine, Ramsay

## Abstract

**Objective:**

The objective of this study was to assess the efficacy and safety of Remimazolam in the context of combined spinal-epidural anesthesia for sedation during orthopedic surgery.

**Methods:**

This randomized controlled trial enrolled patients scheduled for orthopedic surgery under combined spinal-epidural anesthesia (*N* = 80), who were randomly allocated to receive either dexmedetomidine (Group-D) or remimazolam (Group-R). The target sedation range aimed for a Ramsay score of 2–5 or a BIS value of 60–80 to evaluate the effectiveness and safety of remimazolam during sedation.

**Results:**

The time taken to achieve the desired level of sedation was significantly shorter in the remimazolam group compared to the dexmedetomidine group (3.69 ± 0.75 vs. 9.59 ± 1.03; *P* < 0.0001). Patients in the remimazolam group exhibited quicker recovery, fewer intraoperative adverse events, more consistent vital signs, and greater satisfaction at various time points throughout the surgery.

**Conclusion:**

This preliminary study demonstrates that remimazolam tosilate serves as a safe and effective sedative for orthopedic surgery performed under combined spinal-epidural anesthesia, in comparison with dexmedetomidine.

## Introduction

Sedation was initially infrequently employed in orthopedic surgery under combined spinal-epidural anesthesia. However, the potential dangers of uncooperative agitation during orthopedic procedures became evident. The presence of patient anxiety and restlessness posed challenges to successful surgical execution, compromising therapeutic outcomes and leaving patients with distressing experiences [1–3]. Widely utilized sedatives, including propofol, dexmedetomidine, and benzodiazepines, have shown efficacy, though the optimal choice among these options remains uncertain [4, 5].

Desirable sedative attributes encompass user-friendliness, rapid onset, prompt recovery, and minimal lingering sedation [6]. Maintaining cardiovascular function and spontaneous ventilation while enabling purposeful responses to stimuli are essential [7]. Propofol, a potent intravenous sedative, boasts a brief onset and extremely short half-life, facilitating swift recuperation [8, 9]. However, concerns of propofol-related hypotension and respiratory depression persist [5]. The rapid-acting sedative midazolam induces effects within 3–5 min. Nonetheless, its long-lasting metabolite can lead to prolonged post-procedure sedation. Dexmedetomidine, recognized for its sedative and anxiolytic properties, allows reversible sedation [5]. While patients can be easily aroused to lucidity, certain studies associate dexmedetomidine with heightened risks of bradycardia, hypotension, and delayed awakening [2].

Enter remimazolam, a novel ultra-short-acting benzodiazepine [4]. Boasting a swift onset, notably brief metabolic half-life, rapid recovery, and circulatory stability, remimazolam emerges as a promising option [10, 11]. Developed for sedation during therapeutic and diagnostic procedures, induction and maintenance of general anesthesia, and intensive care unit sedation, it is poised to benefit both physicians and patients undergoing orthopedic surgery under combined spinal-epidural anesthesia [12, 13]. Despite remimazolam's potential advantages in this context, research regarding its application in orthopedic surgery under combined spinal-epidural anesthesia remains limited. The present study aims to assess the efficacy and safety of remimazolam for surgical sedation, hypothesizing that remimazolam is non-inferior to dexmedetomidine in terms of sedation effectiveness.

## Materials and methods

### Study design

This single-center, randomized, controlled study obtained approval and registration from the Chinese Clinical Trial Registry (registration no. ChiCTR2200066642, chictr.org.cn, 12/12/2022) and was reviewed by the Ethics Committee of Renmin Hospital (syrmyy2022-80). Written informed consent was acquired from all participating patients or their legal representatives. Eighty adult patients of the American Society of Anesthesiology (ASA) Physical Status I-III, regardless of gender, aged between 40 and 80 years, with a BMI of 18 to 28 kg/m^2^, who were undergoing elective orthopedic surgeries under combined spinal-epidural anesthesia, were selected for inclusion.

The exclusion criteria included patients with major organ diseases, severe physical ailments, cognitive impairment, mental disorders, language or communication deficits, lack of cooperation or informed consent, history of alcohol or drug abuse, long-term use of sedatives, analgesics, or alcohol, alteration of anesthesia during surgery, considerable circulatory fluctuations during surgery, absence of collected efficacy or safety data, and contraindications to spinal anesthesia.

### Sedation protocol

In the Remimazolam group (group-R), an initial bolus of 0.03 mg/kg/min of remimazolam was administered. When patients exhibited undersedation with a Ramsay score of 2–5 and BIS scores ranging from 60 to 80, repeated doses of 0.2–0.5 mg/kg/h of remimazolam were administered to maintain the targeted level of sedation. In the Dexmedetomidine group (group-D), an initial bolus of 0.3 µg/kg of dexmedetomidine was administered. For patients with insufficient sedation, characterized by a Ramsay score of 2–5 and BIS scores ranging from 60 to 80, additional doses of 0.2–1.0 µg/kg/h of dexmedetomidine were administered to sustain the appropriate level of sedation. If the patient did not attain the desired sedation depth 15 min after the initial infusion, an emergency bolus of 0.05 mg/kg of remimazolam was administered. The infusion was halted during the suturing of the skin. No other sedatives or opioids were employed during the surgical procedure.

Heart rate (HR), mean arterial pressure (MAP), oxygen saturation (SpO2), respiratory rate (RR), Ramsay score, and BIS values were recorded at various time points: when patients entered the operating room (T0), when the anesthesia plane stabilized (T1), when sedation achieved effectiveness (T2), when the skin was incised (T3), when the skin was sutured (T4), and when patients were both sedated and awakened (T5). The time to onset of sedation (from the commencement of medication until the BIS value dropped below 80 points or the Ramsay score reached 2 points), the duration of the surgery (from the initiation of the surgical incision until the completion of suturing), and the period of sedation and recovery (when the BIS score exceeded 98 or the Ramsay score equated to 1) were recorded. Additionally, intraoperative hypotension (a decrease in systolic blood pressure of over 20 percent or less than the baseline value), hypoxemia (SpO2 below 90), bradycardia (heart rate less than 50 beats per minute or less than 20 percent of the baseline value), and postoperative somnolence (persistent drowsiness) were documented. Furthermore, patient satisfaction with the overall sedation was assessed.

We did a preliminary study of 20 patients using PASS and calculated the sample size based on the difference in the mean time to sedation onset between the 2 study groups (5.7 ± 1.9 and 7.6 ± 3.0 min), assuming a significance level (α) = 0.05, study power (1-β) = 0.90. The sample size of each group was calculated as 38. To reduce variances, 10% was added to the calculated sample size, which was estimated to be 41 per group for a total sample size of 82. Statistical analyses were carried out using SPSS Version 26.0 (IBM Corp., Armonk, NY, USA) and R software V.4.2.1, in accordance with the established protocol, with statistical significance set at *p* < 0.05. Categorical variables were presented in terms of numbers (percentages) and were subjected to comparison using Pearson’s X^2^ test. Continuous variables were presented as means (standard deviations) or medians (interquartile ranges) and were analyzed using an independent samples t-test or a Mann–Whitney U test following assessment of normality [14, 15].

## Result

### Study population

**Fig. 1:**
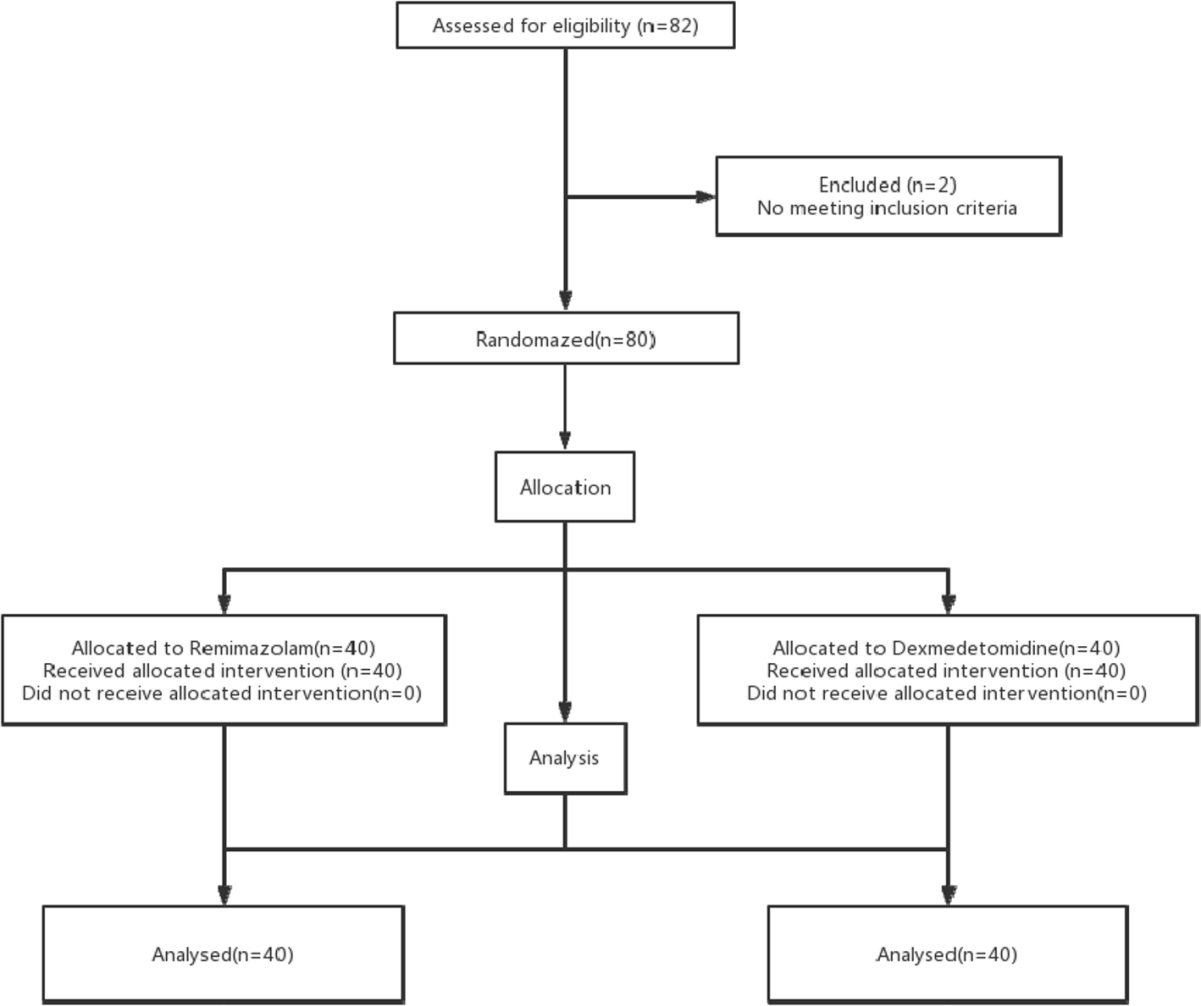
Consolidated standards of reporting trials diagram

The trial assessed 82 patients, two of whom did not adhere to the fasting and drinking guidelines on the day of surgery and were subsequently excluded. Ultimately, a total of 80 patients were included, with 40 individuals randomly allocated to Group-R and 40 to Group-D (Fig. 1).

### Baseline patient characteristics

Demographic data is presented in Table 1. The mean age was 58.77 ± 10.67 years in Group-R and 61.70 ± 12.05 years in Group-D, respectively (*P* = 0.38). While the proportion of female patients was slightly higher in Group-R, this difference was not statistically significant (remimazolam vs dexmedetomidine: 65.0% vs 55.0%, *P* = 0.30). Moreover, no disparities were observed in terms of body mass index, surgery duration, and ASA Physical Status. The average maintenance sedation dose in Group-R was 16.87 ± 4.76 mg, whereas in Group-D, it was 70.09 ± 21.37 µg (Table 1).

**Table 1:** Patient demographics

	Remimazolam(*n* = 40) n(%) or mean ± SD	Dexmedetomidine(*n* = 40) n(%) or mean ± SD	*P*-value
BMI(kg/m^2^)	22.72 ± 2.92	23.18 ± 2.91	0.48
Age(years)	58.77 ± 10.67	61.70 ± 12.05	0.25
Sex, n(%)			0.30
Men	14(35.00%)	18(45.00%)	
Women	26(65.00%)	22(65.00%)	
Operation Time(min)	75.90 ± 29.88	77.20 ± 25.03	0.83
ASA status			0.87
I	11(27.50%)	10(25.00%)	
II	20(50.00%)	19(47.50%)	
III	9(22.50%)	11(27.50%)	

*BMI* Body mass index, *ASA status* American Society of Anesthesiology

Data are count (%), mean (SD)

### Sedation-related outcomes

**Fig. 2:**
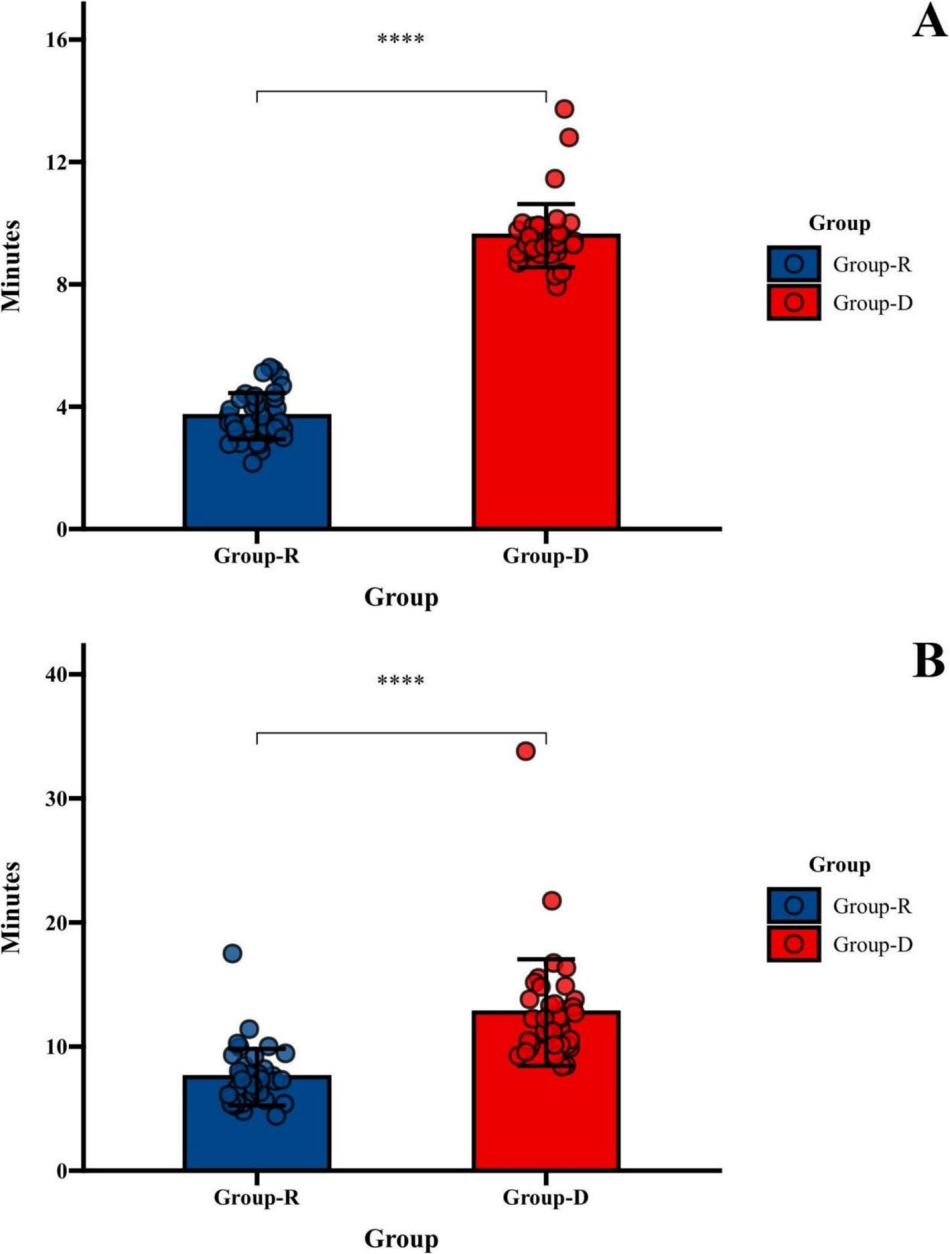
**A** Time to target sedation depth for remimazolam and dexmedetomidine. **B** The time at which remimazolam and dexmedetomidine reached the standard of arousal. ^****^P < 0.0001 relative to Group-D(*n* = 40)

Significant differences were observed in both the time required for sedation and recovery between the two groups. The remimazolam group exhibited a sedation time of 3.69 ± 0.75 min, while the dexmedetomidine group had a sedation time of 9.59 ± 1.03 min (*p* < 0.0001). Correspondingly, the wakefulness time was 7.53 ± 2.28 min for the remimazolam group and 12.74 ± 4.29 min for the dexmedetomidine group (*p* < 0.0001) (Fig. 2).

### Relevant vital signs during sedation

**Fig. 3:**
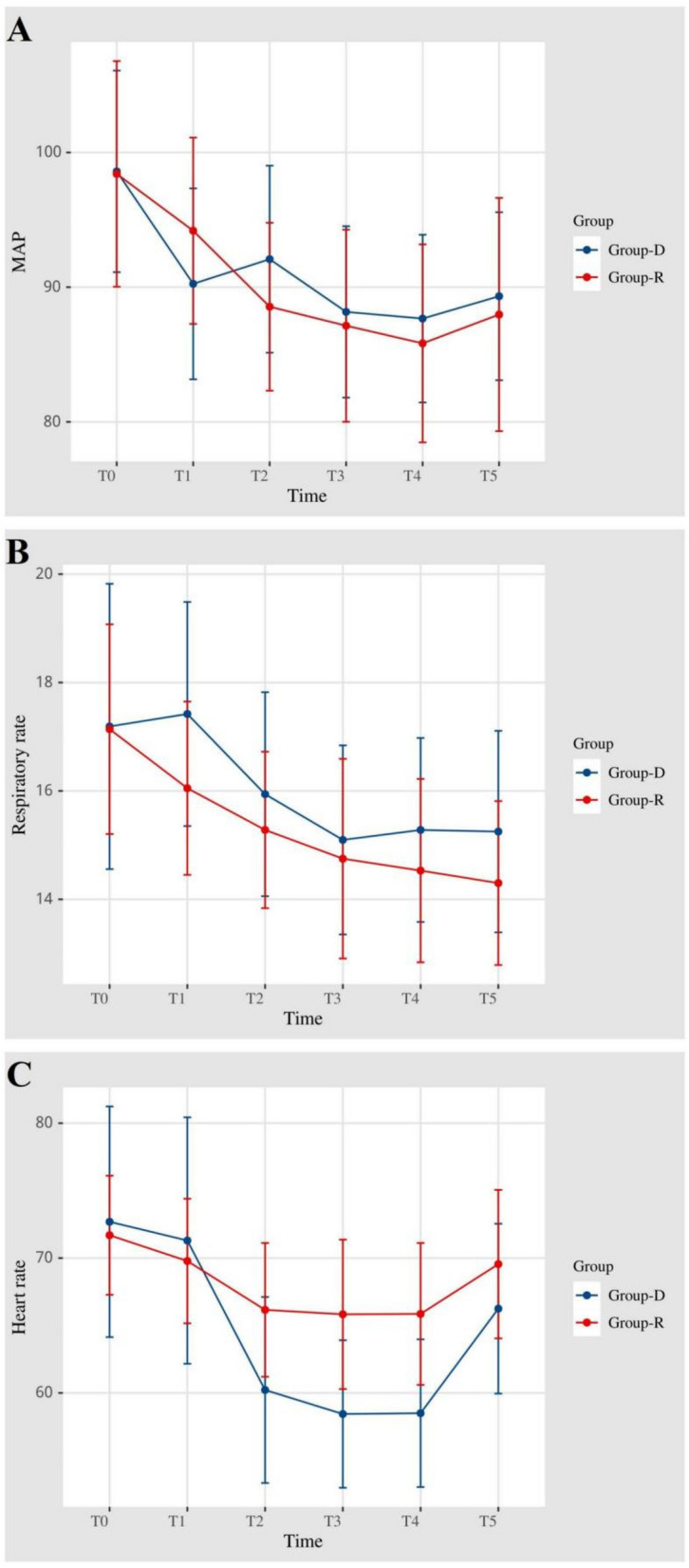
The related life parameters of remimazolam and dexmedetomidine at different time points during sedation in combined spinal-epidural anesthesia for orthopaedic surgery. **A** The mean arterial pressure was measured at T 0, T 1, T 2, T 3, T 4 and T 5, respectively. **B** The respiratory rate was measured at T 0, T 1, T 2, T 3, T 4 and T 5, respectively. **C** The heart rates were measured at T 0, T 1, T 2, T 3, T 4 and T 5, respectively

Vital signs during sedation are depicted in the figure. Main parameters such as mean arterial pressure (MAP), heart rate (HR), and respiratory rate (RR) were closely monitored during sedation in both groups. No significant differences were noted in mean arterial pressure and respiratory rate at the six time points. However, a notable disparity in heart rate was observed at T2 (*P* = 0.02), T3 (*P* = 0.002), and T4 (*P* = 0.001) (Fig. 3).

### Adverse events and satisfaction with sedation

Within the remimazolam group, eleven patients encountered adverse events during the sedation procedure, in contrast to twenty-four patients within the dexmedetomidine group (remimazolam vs dexmedetomidine: Transient hypotension, 15.00% vs 10.00%; Desaturation, 10.00% vs 2.50%; Bradycardia, 7.50% vs 52.50%; Lethargy, 2.50% vs 17.50%; *P* = 0.003). Among these cases, four patients within the remimazolam group reported experiencing two or more adverse events, in comparison to eight patients within the dexmedetomidine group (Table 2).

**Table 2:** Major adverse reactions occurred during sedation

	Remimazolam(*n* = 40)	Dexmedetomidine(*n* = 40)	*P*-value
Total	40	40	
Normal events	29(72.50%)	16(40.00%)	
Adverse events	11(27.50%)	24(60.00%)	0.003
Transient hypotension	6(15.00%)	4(10.00%)	
Desaturation	2(5.00%)	1(2.50%)	
Bradycardia	3(7.50%)	21(52.50%)	
Lethargy	1(2.50%)	7(17.50%)	

Data are presented as n (%)

**Fig. 4:**
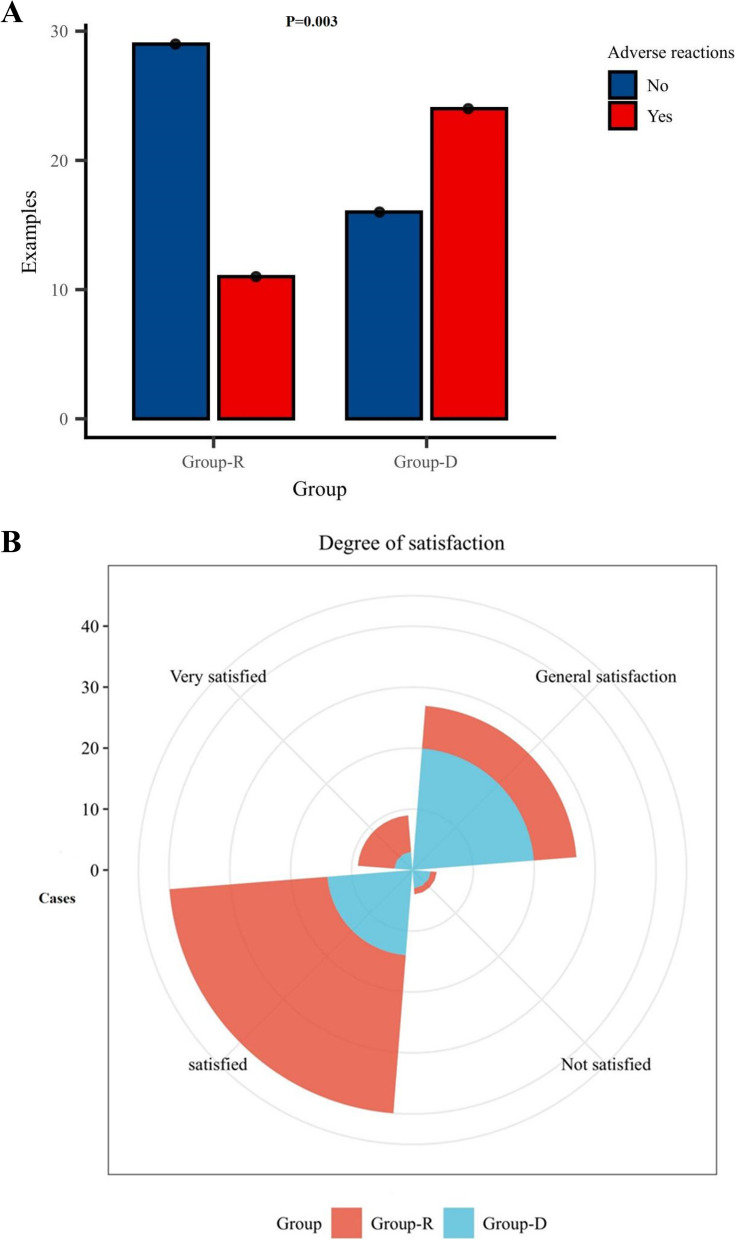
**A** Adverse reactions to remimazolam and dexmedetomidine during intraoperative sedation

In both cohorts, a majority of patients expressed contentment with the sedation administered during the procedure (remimazolam vs dexmedetomidine: highly satisfied, 12.50% vs 7.50%; moderately satisfied, 65.00% vs 32.50%; generally satisfied, 20.00% vs 52.50%; dissatisfied, 2.50% vs 7.50%; *P* < 0.001) (Fig. 4A, B).

## Discussion

This trial yielded three significant findings. Firstly, the desired level of sedation was successfully attained with remimazolam during lower extremity orthopedic surgery. Secondly, the drug exhibited a favorable safety profile, characterized by a lower incidence of adverse events compared to dexmedetomidine. Thirdly, remimazolam demonstrated a rapid onset along with prompt wakefulness, contributing to increased patient satisfaction.

Being an ultra-short-acting benzodiazepine sedative, remimazolam possesses attributes aligning with an ideal sedative profile [16]. These qualities encompass rapid onset, brief duration, swift recovery, and reduced occurrence of adverse reactions [6]. This makes it a promising candidate for continuous sedation during surgical procedures [3]. Past research has established the efficacy of remimazolam in sedation for painless gastroscopy, ICU procedural interventions, and bronchoscopy [8, 9, 17]. It is noteworthy that escalating concentrations of remimazolam can induce profound sedation, leading to short-term hypoventilation and the potential for hypoxemia and injection-related discomfort [18]. Such circumstances necessitate deep sedation for procedures involving strong stimuli, like invasive therapies [19]. However, moderate sedation can alleviate negative emotional states, promote patient tranquility and cooperation, thereby achieving optimal sedative effects and mitigating adverse reactions during lower limb orthopedic surgery under combined spinal-epidural anesthesia [20].

This investigation revealed the effectiveness of remimazolam tosilate for injection and dexmedetomidine in achieving sedation using combined spinal-epidural anesthesia for lower extremity orthopedic surgery. Both agents facilitated the attainment of appropriate sedation levels. Notably, the sedation success rate with remimazolam was comparable to dexmedetomidine. This aligns with earlier findings wherein effective sedation was achieved within around 3 min for remimazolam and 10 min for dexmedetomidine. The respective durations for full recovery from sedation were approximately 9 min and 14 min.

In terms of maintaining hemodynamic stability during sedation, remimazolam exhibited comparable efficacy to dexmedetomidine. Prior studies have indicated that dexmedetomidine, at higher plasma concentrations, induces transient bradycardia, which can be rectified through reduced infusion rates [2, 21]. In line with these observations, temporary bradycardia occurred in the dexmedetomidine group during desired sedation depths, followed by gradual recovery [22, 23]. Notably, heart rate fluctuations were more stable in the remimazolam group.

In this study, adverse reactions were observed in 11 (27.5%) patients in the remimazolam group and 24 (60%) patients in the dexmedetomidine group. A noteworthy discrepancy in the occurrence of adverse events between the two groups was evident. The remimazolam group primarily experienced transient hypotension (15%), whereas bradycardia was the predominant adverse event in the dexmedetomidine group (52.5%). The study also identified no significant disparity in oxygenation and respiratory events. This could be attributed to patient cooperation and initial lateral placement of the head and neck, which mitigated the risk of airway obstruction.

Given the potential for fear, anxiety, and adverse emotions, combined spinal-epidural anesthesia is crucial for ensuring appropriate sedation during orthopedic surgery. The benefits of remimazolam, including reduced complications and retrograde amnesia, contributed to enhanced patient satisfaction.

However, certain limitations warrant consideration. Firstly, this study did not exclude confounding factors affecting hemodynamics, such as surgical position, procedure type, and bleeding volume. Secondly, data collection was confined to the operating room, without post-operative follow-up upon patients' return to the ward. Lastly, the scope of this study solely encompassed sedation for orthopedic surgery under combined spinal-epidural anesthesia. Future research could extend to investigating analgesic approaches for diverse surgical procedures under regional anesthesia.

## Conclusion

Remimazolam tosilate proves to be an efficacious sedative for orthopedic surgery conducted under combined spinal-epidural anesthesia. Its rapid onset and swift recovery properties are particularly noteworthy, contributing to the reduction of adverse reactions and the enhancement of patient satisfaction. This is achieved while maintaining an optimal level of sedation depth.

## Data Availability

The datasets used and/or analysed during the current study available from the corresponding author on reasonable request.
